# Maternal and child genetic liability for smoking and caffeine consumption and child mental health: an intergenerational genetic risk score analysis in the ALSPAC cohort

**DOI:** 10.1111/add.15521

**Published:** 2021-05-10

**Authors:** Laura Schellhas, Elis Haan, Kayleigh E. Easey, Robyn E. Wootton, Hannah M. Sallis, Gemma C. Sharp, Marcus R. Munafò, Luisa Zuccolo

**Affiliations:** ^1^ School of Psychological Science University of Bristol Bristol UK; ^2^ MRC Integrative Epidemiology Unit at the University of Bristol Bristol UK; ^3^ Department of Population Health Sciences, Bristol Medical School University of Bristol Bristol UK; ^4^ NIHR Biomedical Research Centre at the University Hospitals Bristol NHS Foundation Trust and the University of Bristol Bristol UK; ^5^ Centre for Academic Mental Health, Bristol Medical School University of Bristol Bristol UK; ^6^ Bristol Dental School University of Bristol Bristol UK

**Keywords:** ALSPAC, caffeine, genetic risk score, intergenerational effects, mental health, tobacco

## Abstract

**Background and aims:**

Previous studies suggest an association between maternal tobacco and caffeine consumption during and outside of pregnancy and offspring mental health. We aimed to separate effects of the maternal environment (intrauterine or postnatal) from pleiotropic genetic effects.

**Design:**

Secondary analysis of a longitudinal study. We (i) validated smoking and caffeine genetic risk scores (GRS) derived from published genome‐wide association study (GWAS) for use during pregnancy, (ii) compared estimated effects of maternal and offspring GRS on childhood mental health outcomes and (iii) tested associations between maternal and offspring GRS on their respective outcomes.

**Setting:**

We used data from a longitudinal birth cohort study from England, the Avon Longitudinal Study of Parents and Children (ALSPAC).

**Participants:**

Our sample included 7921 mothers and 7964 offspring.

**Measurements:**

Mental health and non‐mental health phenotypes were derived from questionnaires and clinical assessments: 79 maternal phenotypes assessed during and outside of pregnancy and 71 offspring phenotypes assessed in childhood (<10 years) and adolescence (11–18 years).

**Findings:**

The maternal smoking and caffeine GRS were associated with maternal smoking and caffeine consumption during pregnancy (2nd trimester: P_smoking_ = 3.0 × 10^−7^, P_caffeine_ = 3.28 × 10^−5^). Both the maternal and offspring smoking GRS showed evidence of association with reduced childhood anxiety symptoms (β_maternal_ = −0.033; β_offspring_ = −0.031) and increased conduct disorder symptoms (β_maternal_ = 0.024; β_offspring_ = 0.030), after correcting for multiple testing. Finally, the maternal and offspring smoking GRS were associated with phenotypes related to sensation seeking behaviours in mothers and adolescence (e.g. increased symptoms of externalising disorders, extraversion and monotony avoidance). The caffeine GRS showed weaker evidence for associations with mental health outcomes.

**Conclusions:**

We did not find strong evidence that maternal smoking and caffeine genetic risk scores have a causal effect on offspring mental health outcomes. Our results confirm that the smoking genetic risk scores also captures liability for sensation seeking personality traits.

## Introduction

Smoking and caffeine consumption often co‐occur [1] and are associated with mental health problems and other substance use behaviours [2, 3]. There is some evidence that smoking is a causal risk factor for mental health problems, such as depression and schizophrenia [4, 5]; however, the relationship between caffeine and mental health is less clear, and possibly difficult to disentangle from smoking because the two often co‐occur [3, 6]. In addition to associations between smoking, caffeine and mental health outcomes within individuals, observational research suggests that prenatal maternal consumption of tobacco and caffeine could have an intergenerational effect on offspring’s mental health [7, 8, 9, 10].

Using conventional epidemiological methods alone, it is difficult to ascertain whether prenatal tobacco and caffeine exposure causally affect offspring mental health outcomes [11, 12]. Not only do mothers and offspring share a similar environment (such as socioeconomic position), they also share, on average, 50% of their segregating genetic variation. Because of this shared genetic and environmental confounding, it is difficult to disentangle the effect of maternal substance use on offspring mental health from those of offspring’s own substance use.

The association between maternal prenatal smoking and internalising problems in children is less extensively researched compared to associations with externalising problems, and existing evidence is mixed [9, 13, 14, 15]. Many studies report a positive association between prenatal smoking and offspring’s externalising problems [7, 16, 17, 18], which could reflect a potential intrauterine effect of smoking. However, results vary when adopting different methods to account for shared environmental and genetic confounders [16]. For example, studies using negative control designs and sibling comparisons have found inconclusive evidence for a causal intrauterine effect [16, 17, 19, 20, 21]. In fact, study designs adjusting for shared genetic factors between mother and offspring have concluded that genetic factors explain associations between maternal prenatal smoking and externalising problems in offspring [22]. This literature highlights the complexity of the nature of associations between pregnancy exposures and offspring mental health, and the importance of disentangling shared genetic and environmental confounders to understand whether a true causal effect exists.

Using genetic risk scores (GRS) as proxies for smoking or caffeine consumption can, in principle, reduce bias from confounding [23]. However, when investigating intergenerational effects, this approach may lead to spurious results for several reasons [24]. First, the genetic variants used in the GRS have mostly been identified and validated in non‐pregnant adult populations and therefore might not predict behaviour during pregnancy [24, 25, 26]. Second, offspring’s own smoking or caffeine consumption may confound associations because mothers pass on their genetic predisposition for smoking or caffeine consumption to their children. Consequently, when offspring’s mental health outcomes are assessed at an age where offspring are likely to have started smoking or drinking caffeine themselves, offspring’s own consumption may cause offspring’s mental health problems. Third, an association between maternal GRS and offspring mental health outcomes may reflect a shared genetic liability for smoking or caffeine consumption and mental health outcomes (pleiotropy) instead of a causal effect of the exposure. Given the shared genetics between parents and offspring, intergenerational GRS analyses should control for both offspring GRS and paternal GRS to avoid collider bias, but often it is not possible because of the limited availability of data on mothers, fathers and offspring, and limited sample size in many cohort studies [14].

**Figure 1:**
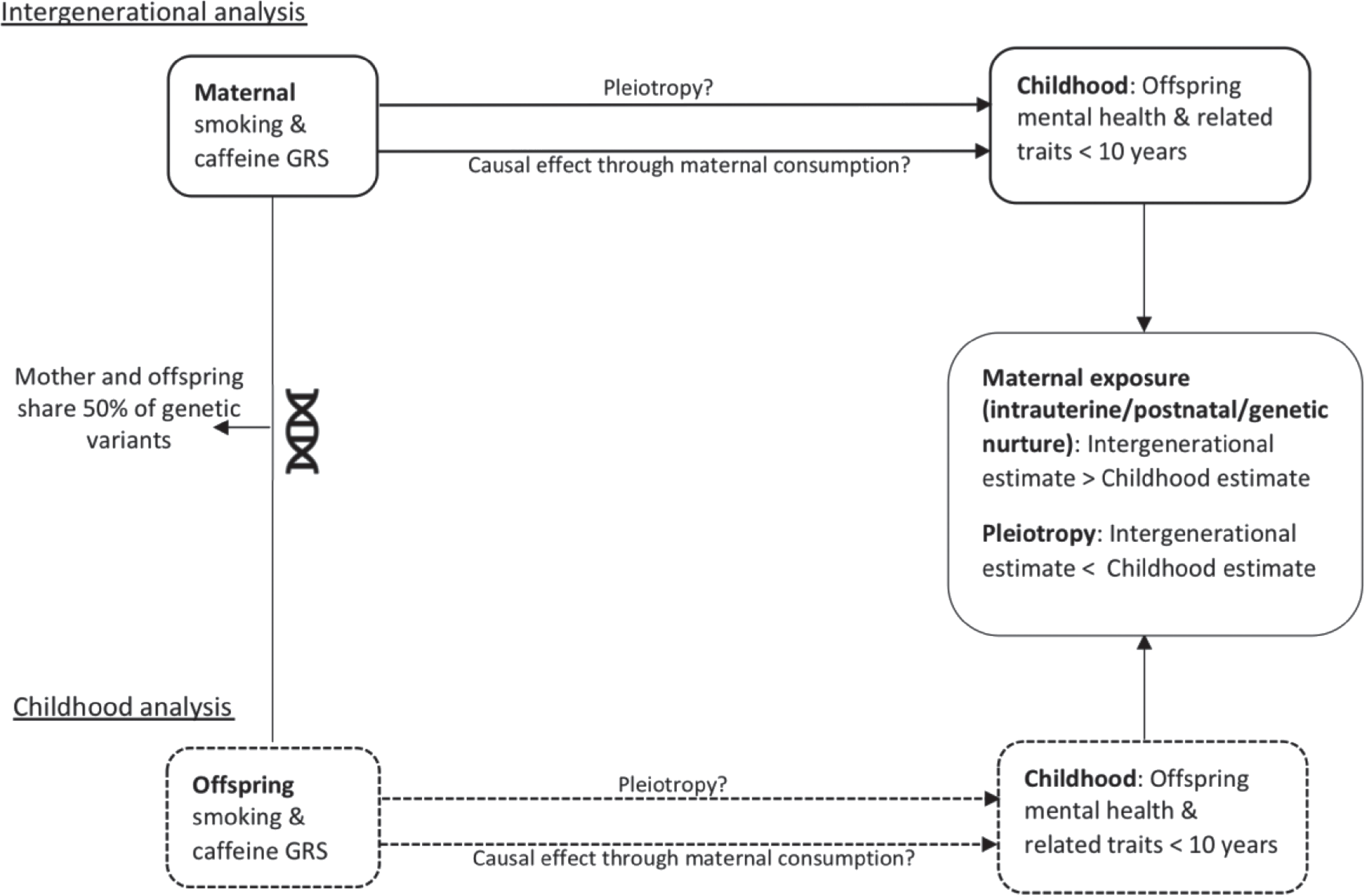
Analyses of aim 2: comparison of intergenerational and childhood analysis results to disentangle maternal environmental from pleiotropic effects. Design overview of the analyses of aim 2, which compares the intergenerational analysis (top) and the childhood analysis (bottom). A larger effect estimate in the intergenerational compared to the childhood analysis would reflect a causal effect of caffeine/smoke exposure through the maternal environment (intrauterine/postnatal/genetic nurture). A larger effect estimate in the childhood compared to the intergenerational analysis would reflect pleiotropic association of the genetic risk scores with mental health

In this study, we aimed to elucidate the effects of maternal prenatal smoking and caffeine consumption on offspring mental health, using data from a multi‐generational cohort study from England, the Avon Longitudinal Study of Parents and Children (ALSPAC) [27]. We had two specific aims: (i) to validate the smoking and caffeine GRS during pregnancy (in mothers) and adolescence (in offspring) and (ii) to estimate the effect of maternal smoking and caffeine consumption on offspring mental health. The second aim was achieved by first estimating the association between maternal smoking and caffeine GRS with offspring mental health outcomes during childhood (before age 10 years when children are unlikely to start smoking or consuming higher level of caffeine themselves; childhood GRS analysis, Fig. 1), and then comparing the effect of mothers GRS and offspring GRS on offspring mental health to disentangle pleiotropic from potential causal associations (intergenerational GRS analysis, Fig. 1).

## Methods

### Design

A visual overview of the study design can be found in Fig. 1. Given the shared genetic material between mothers and offspring, we expect pleiotropic associations to be reflected by a larger estimated effect of the offspring GRS on offspring mental health, compared to the estimated effect of the maternal GRS (childhood GRS analysis). Following the same reasoning, a larger estimated effect of the maternal GRS on offspring mental health (relative to the estimated effect of the offspring GRS) would provide more evidence to support a causal effect of maternal behaviour on offspring mental health (intergenerational GRS analysis).

### Study population

The ALSPAC is a prospective longitudinal cohort study where the initial number of pregnancies enrolled is 14 541 and of these initial pregnancies, there were a total of 14 676 fetuses, resulting in 14 062 live births and 13 988 children who were alive at 1 year of age. When the oldest children were approximately 7 years of age, an attempt was made to bolster the initial sample with eligible cases who had failed to join the study originally, resulting in an additional 913 children being enrolled. The total sample size for analyses using any data collected after the age of seven is therefore 15 454 pregnancies, resulting in 15 589 fetuses, of these 14 901 were alive at 1 year of age [27, 28, 29]. The ALSPAC study was approved by the ALSPAC Ethics and Law Committee and the Local Research Ethics Committees and informed consent for the use of data collected via questionnaires and clinics was obtained from participants. The study website contains details of all the data that is available through a fully searchable data dictionary and variable search tool (http://www.bristol.ac.uk/alspac/researchers/our‐data/).

### Phenotype data

Mental health phenotypes were selected from questionnaires and clinical assessments. Besides mental health phenotypes, some non‐mental health phenotypes were also included that were selected based on evidence in the literature indicating high comorbidity with mental health problems (e.g. alcohol, cannabis, other drugs, personality, body mass index (BMI), sleep, socio‐economic variables). To validate the GRS, we derived phenotypes to describe caffeine consumption and smoking behaviours. Offspring assessment points were grouped into ‘childhood’ (age 7–11 years) and ‘adolescence’ (age 12–18 years). Maternal assessment points were grouped into ‘during pregnancy’ (8, 18 and 32 weeks of gestation) and ‘outside of pregnancy’, which included phenotypes assessed pre‐ and/or post‐pregnancy. Outcomes assessed within the first four years after pregnancy were excluded, because the transition to parenthood may influence mental health temporarily [30] and mothers may be more likely to be pregnant again. In total we included 71 phenotypes for offspring (childhood and adolescence) and 79 phenotypes for mothers (during and outside of pregnancy). Table 1 gives an overview of phenotypes included in the intergenerational and childhood GRS analyses across timepoints. A complete list of phenotypes is given in **Supporting information Table** **S1** **.** More details about the phenotype selection and assessment can be found in Supporting Information.

**Table 1:** Availability of phenotypes included in the intergenerational and childhood analyses across sub‐populations.

*Measures*	*Timepoints*			
	*Offspring*		*Mothers*	
	*Childhood (age <10)*	*Adolescence (age 12–18)*	*Outside of pregnancy (pre/post‐pregnancy)*	*During pregnancy*
Mental health				
Emotional problems				
Depression symptoms	x	x	x	x
Anxiety symptoms	x	x	x	x
Specific phobia	x	x		
Behavioural problems				
ODD symptoms	x	x	Personality measures (extraversion, anger, impulsivity)	
Conduct disorder symptoms	x	x		
ADHD symptoms	x	x		
Total behavioural difficulties	x	x		
Neuro‐developmental				
Autism (lifetime diagnosis)	x			
Other				
Handedness (negative control)	x			
IQ	x	x	Only education and SEP	Only education and SEP
Number of stressful life events	x	x	x	x
BMI	x	x	x	Only image perception and physical activity
Sleep initiation	x	x		
Sleep maintenance	x	x		
Hours of sleep (duration)	x	x	x	
Overall caffeine intake	x	x	x	x

ADHD = attention deficit hyperactivity disorder; BMI = body mass index; IQ = intelligence quotient; ODD = oppositional defiant disorder; SEP = socioeconomic position.

### Genetic risk scores

In ALSPAC, genome‐wide SNP data were available for 8237 children and 8196 mothers (detailed information about genotyping can be found in Supporting Information). After removing individuals who withdrew their consent or did not pass quality control, GRS could be generated for 7964 children and 7921 mothers (see Supporting Information for more details [31]). The genome‐wide association study (GWAS) and Sequencing Consortium of Alcohol and Nicotine use (GSCAN; *n* = 1.2 million [25]) identified 378 single nucleotide‐polymorphisms (SNPs) associated with smoking initiation that were conditionally independent at the genome‐wide significance level (*P* < 5 × 10^−8^). Smoking initiation was defined as being an ‘ever’ vs. ‘never’ smoker where an ‘ever’ smoker had to have either smoked 100 cigarettes in their lifetime and/or smoked regularly every day for at least a month. Of the 378 genome‐wide significant SNPs, 356 were available in ALSPAC [25]. Considering that smoking is a complex behaviour, of which initiation is only one part, we also generated a GRS for lifetime smoking. The lifetime smoking score also captures smoking heaviness (as well as smoking duration and cessation) but is derived in the entire population comprising both smokers and non‐smokers and therefore is more suitable for use in unstratified samples [4]. The GWAS of lifetime smoking based on the United Kingdom (UK) Biobank data (*n* = 462 690) identified 126 independent loci (*P* < 5 × 10^−8^), which were all available in ALSPAC. The Coffee and Caffeine Genetics Consortium found 8 SNPs to be independently associated with cups of coffee consumed per day at the genome‐wide level of significance (*n* = 91 462 [26]), which were all available in ALSPAC. These SNPs have also been found to be associated with caffeine use from other caffeinated beverages [32, 33].

We created weighted genetic risk scores using independent genome wide significant hits (*P* < 5 × 10^−8^) and their effect estimates as reported in the discovery GWAS for each of our exposures. These GRS were derived using Plink v1.9 and standardised before use in analyses. Because our GRS were based on discovery GWAS that only report independent variants [4, 25, 26], clumping or pruning were not necessary [34].

### Statistical analysis

The statistical analysis plan for this secondary analysis of study data was not preregistered and the results should be considered exploratory. All analyses were performed using Stata v15 [35]. The following linear and logistic regression analyses were conducted to test associations with the smoking and caffeine GRS: (i) maternal GRS with smoking and caffeine phenotypes in mothers during pregnancy to validate the GRS (aim 1); (ii) maternal and offspring GRS with childhood phenotypes (<10 years) for investigating intergenerational effects (Fig. 1) (aim 2); and (iii) (supplementary analyses) maternal and offspring GRS with their own phenotypes in mothers (during and outside of pregnancy) and offspring (adolescence) to confirm GRS associations with relevant substance use behaviours as a positive control and gain more information about mental health associations at later times in development. Analyses were adjusted for age, offspring sex and the first 10 ancestry‐informative principal components based on the ALSPAC genome‐wide data. We restricted our sample to singletons or one individual from a twin pair and to individuals of European ancestry. The maximum sample size available in childhood was 6156 (4974 in adolescence) and 7269 during pregnancy (7199 outside of pregnancy). To avoid limiting our sample size further, and to reduce the risk of selection bias, we did not restrict our analyses to only mother‐offspring pairs with complete genotype data. We compared mother‐offspring pairs where either mother or offspring have genotype data but not both with respect to smoking, caffeine and socio‐demographic variables. This comparison is shown in **Supporting information Table** **S2** **.**

### Multiple testing

Multiple testing was accounted for by running Monte Carlo permutation testing with 1000 repetitions. These *P* values are presented in the text. We also compared these results with a more stringent Bonferroni correction. However, given the high degree of correlation between our phenotypes, this correction is likely to be overly conservative. Evidence for association was considered strongest for phenotypes that also survived Bonferroni correction (all results are available in the Supporting Information).

## Results

### Maternal smoking and caffeine consumption

In our sample, 51% of mothers reported having ever smoked a cigarette in their lifetime and 25% reported smoking during the first trimester of pregnancy. Mothers’ median caffeine consumption outside of pregnancy (97 months post‐pregnancy) was 168 milligrams of caffeine a day (mg/day; interquartile range [IQR]: 108–252). During pregnancy, mothers reported lower caffeine consumption with a median of 138 mg/day (IQR: 81 to 215) during the 2nd trimester and 135 mg/day (IQR: 71–216) during the 3rd trimester. Compared to mothers who did not report smoking, mother who smoked reported consistently more caffeine consumption during (2nd trimester: 64 mg/day more caffeine, 3rd trimester: 75 mg/day more caffeine) and outside of pregnancy (8 years post‐pregnancy: 30 mg/day more caffeine).

### Validation of GRS during pregnancy

The GRS for smoking initiation and lifetime smoking were positively associated with maternal smoking phenotypes during pregnancy and explained 1–5% of variance in smoking phenotypes during and outside of pregnancy (Table 2 and **Supporting information Table** **S3** **)**. The GRS for caffeine consumption was positively associated with total caffeine and caffeinated tea and coffee consumption during pregnancy, but not with cola consumption (Table 3). The caffeine GRS explained 0.2–0.4% of variance in caffeine phenotypes during pregnancy and 0.2–1% of variance outside of pregnancy (Table 2).

**Table 2:** Associations between smoking initiation GRS and smoking phenotypes in mothers (during and outside of pregnancy) and offspring in adolescence.

	Phenotype	Effect estimate	Effect size^a^	95% CI	P value	Sample size	Adj. R^2^ ^b^
Mothers							
Outside of pregnancy	Mother has ever smoked	OR	1.40	1.33, 1.48	1.24 × 10^–8^	7194	0.03
	Number of cigarettes smoked before pregnancy	Beta	0.15	0.08, 0.22	3.81 × 10^–5^	3426	0.05
Pregnancy– 18 weeks gestation	Tobacco smoked in first 3 months of pregnancy	OR	1.35	1.23, 1.44	3.0 × 10^–7^	7237	0.05
	Mother cut down smoking	OR	1.33	1.25, 1.42	5.89 × 10^–7^	7269	0.03
	Mother stopped smoking during pregnancy	OR	0.98	0.88, 1.11	0.771	1863	0.01
Offspring							
Adolescence– 14 years	Smoked at age 14 years	OR	1.18	1.09, 1.28	6.50 × 10^–4^	4145	0.03
	Smoked more than 20 cigarettes	OR	1.19	1.03, 1.38	0.024	1058	0.03
	Age 1st smoked a cigarette	Beta	0.001	−0.04, 0.04	0.953	1064	0.01
Adolescence– 18 years	Ever smoked a whole cigarette	OR	1.26	1.15, 1.37	1.09 × 10^–4^	2402	0.02
	Number of cigarettes smoked in lifetime	Beta	0.19	0.10, 0.2	4.24 × 10^–5^	1144	0.01

GRS = genetic risk scores.

^a^ Reflects the average change in the outcome that is associated with a one SD increase in the GRS. For binary outcomes, this will be the OR (e.g. mother's odds of ever smoking are 1.4 times compared to not smoking), for continuous outcomes it represents the average unit change (e.g. 0.15 cigarettes smoked).

^b^ For the logistic regression models pseudo R^2^ is reported.

**Table 3:** Associations between caffeine GRS and daily caffeine intake in mothers (during and outside of pregnancy) and offspring (age 8 and 13 years).

	*Daily caffeine intake phenotype*	*Effect size* ^a^ (*beta*)	*95% CI*	*P value*	*Sample size*	*R* ^2^ ^b^
Mothers						
Outside of pregnancy	Total (coffee, tea and cola)	9.89	6.34, 13.44	4.97 × 10–8	4783	0.01
	Coffee	0.03	0.01, 0.06	0.009	4655	0.003
	Tea	0.07	0.03, 0.10	1.01 × 10–4	4632	0.01
	Cola	0.01	−0.01, 0.03	0.332	4670	0.002
Pregnancy – 18 weeks gestation	Total (coffee, tea and cola)	5.85	3.09, 8.61	3.28 × 10–5	7220	0.004
	Coffee	0.02	0.01, 0.04	0.011	7198	0.002
	Tea	0.02	0.01, 0.04	0.007	7189	0.002
	Cola	−0.001	−0.02, 0.01	0.890	7185	0.002
Pregnancy – 32 weeks gestation	Total (coffee, tea and cola)	6.32	3.74, 8.89	1.56 × 10–6	6767	0.01
	Coffee	0.03	0.01, 0.04	0.01	6596	0.002
	Tea	3.42	1.80, 5.04	3.65 × 10–5	6608	0.004
	Cola	−0.01	−0.03, 0.01	0.278	6500	0.002
Offspring						
Childhood – age 8 years	Total (coffee, tea and cola)	0.01	−0.01, 0.03	0.377	4589	0.002
	Coffee	0.01	−0.06,0.08	0.845	254	0.02
	Tea	0.18	−1.52, 1.88	0.836	1475	0.004
	Cola	0.003	−0.02, 0.03	0.829	4551	0.002
Adolescence – age 13 years	Total (coffee, tea and cola)	0.01	−0.03, 0.05	0.670	3405	0.004
	Coffee	0.03	−0.02, 0.08	0.271	467	0.05
	Tea	0.89	−0.35, 2.13	0.161	1933	0.004
	Cola	−0.02	−0.05, 0.02	0.424	2411	0.01

GRS = genetic risk scores;

^a^ Reflects the average change in the outcome that is associated with a one SD increase in the GRS. For continuous outcomes it represents the average unit change (e.g. a one SD increase in GRS is associated with mothers consuming 9.89 mg/day more caffeine outside of pregnancy). For transformed variables, it represents the average quantile or quartile change (e.g. a one SD change in GRS is associated with a 0.03 quantile mg/day increase in coffee consumption outside of pregnancy, Supporting Information).

^b^ For the logistic regression models pseudo R^2^ is reported.

### Comparison of intergenerational and childhood smoking initiation GRS analyses

#### Intergenerational GRS analyses

**Figure 2:**
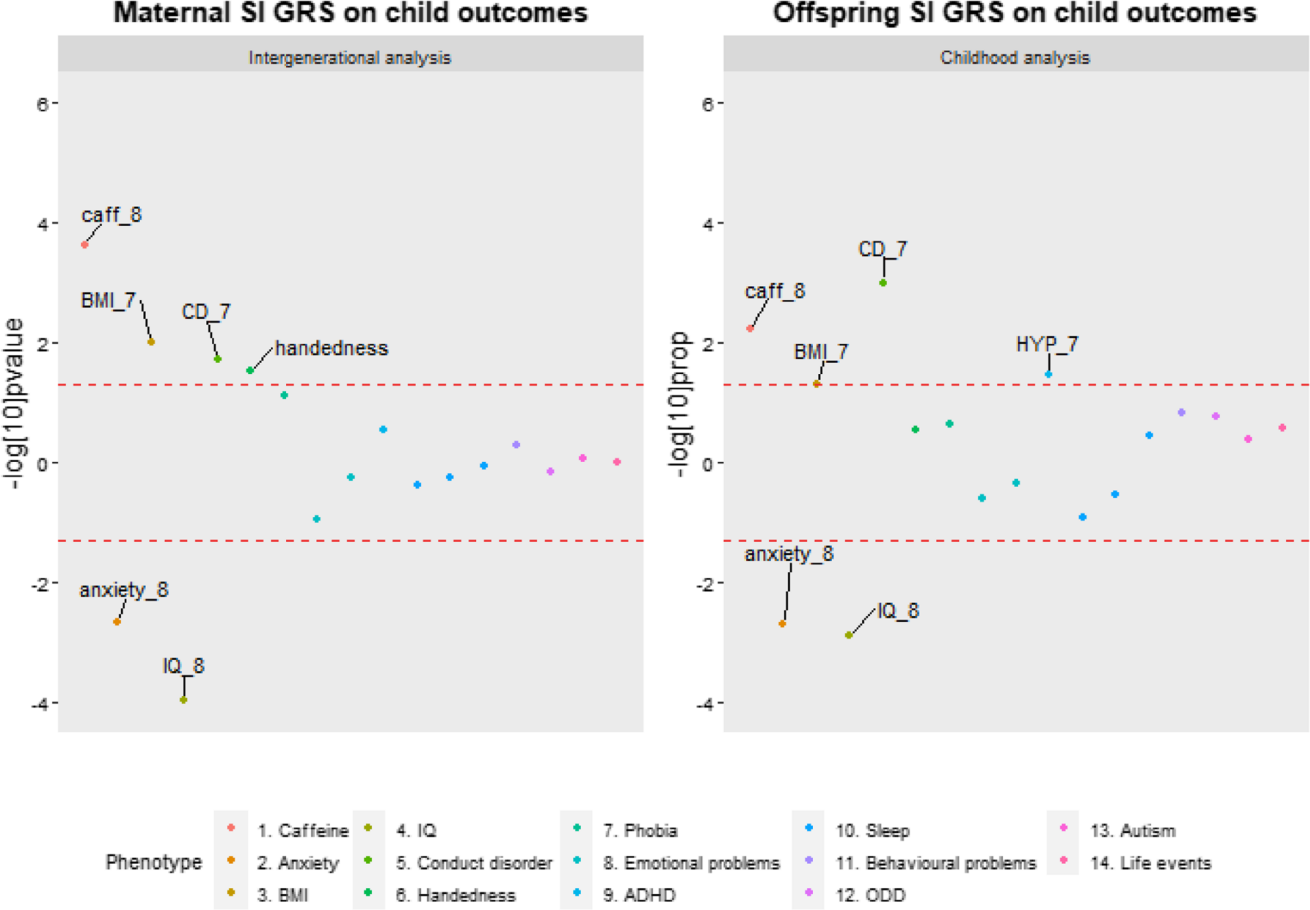
Comparison of phenotype associations with the smoking initiation genetic risk scores (SI GRS) in the intergenerational and childhood analysis. Points outside the lines had a permutation corrected *P* value < 0.05. Points above the upper line represent positive associations and points below the lower line represent negative associations. caff_8 = Total caffeine consumption at age 8. BMI_7 = BMI at age 7. CD = conduct disorder at age 7. anxiety_8 = Anxiety at age 8. IQ_8 = IQ at age 8. HYP_7 = hyperactivity at age 8

Of 17 childhood mental health phenotypes, the strongest evidence of association was observed for reduced anxiety symptoms (P_perm_ = 0.002) and increased conduct disorder symptoms (P_perm_ = 0.021). Of the non‐mental health phenotypes, the strongest associations were found for lower intelligence quotient (IQ) (P_perm_ = 0.02), higher overall caffeine consumption (P_perm_ = <0.001) and body mass index (BMI) (P_perm_ = 0.001) as well as the likelihood of being left‐handed (P_perm_ = 0.012), which was included as a negative control phenotype (because we would not expect a causal intrauterine effect of maternal smoking or caffeine on handedness). The only associations to survive Bonferroni correction (*P* < 0.003) were that of maternal smoking GRS with offspring's anxiety symptoms and offspring's caffeine consumption (Fig. 2, Table 4).

**Table 4:** Associations between the maternal and offspring smoking initiation GRS and offspring phenotypes <10 years.

*Phenotype*	*Effect estimate*	*Intergenerational analyses*						*Childhood analyses*					
		*Regression analyses*			*Permutation testing*			*Regression analyses*			*Permutation testing*		
		*Effect size*	*95% CI*	*P value*	*95% CI*	*P value*	*Sample size*	*Effect size*	*95% CI*	*P value*	*95% CI*	*P value*	*Sample size*
1. Total caffeine	Beta	0.045	0.021, 0.068	<0.001	<0.001, 0.004	<0.001	4067	0.032	0.010, 0.055	0.005	0.002, 0.013	0.006	4589
2. Anxiety	Beta	−0.033	−0.053, −0.012	0.002	<0.001, 0.007	0.002	4993	−0.031	−0.051, −0.010	0.003	<0.001, 0.007	0.002	5355
3. BMI	Beta	0.076	0.018, 0.135	0.010	0.007, 0.022	0.013	5032	0.050	<0.001, 0.101	0.051	0.036, 0.063	0.048	5799
4. IQ	Beta	−0.592	−1.049, −0.134	0.011	0.009, 0.026	0.016	4675	−0.735	−1.183, −0.287	0.001	<0.001, 0.004	<0.001	5295
5. Conduct disorder	Beta	0.024	0.004, 0.044	0.019	0.013, 0.032	0.021	5012	0.030	0.012, 0.049	0.001	<0.001, 0.006	0.001	5326
6. Handedness	OR	1.114	1.012, 1.225	0.030	0.006, 0.021	0.012	4849	1.045	0.954, 1.145	0.315	0.263, 0.320	0.291	5403
7. Specific phobia	OR	1.322	0.964, 1.813	0.078	0.042, 0.071	0.055	5100	1.182	0.881, 1.587	0.241	0.199, 0.252	0.225	5470
8. Emotional problems	Beta	−0.016	−0.037, 0.004	0.117	0.106, 0.148	0.126	5139	−0.011	−0.031, 0.009	0.267	0.236, 0.291	0.263	5459
9. ADHD	Beta	0.016	−0.013, 0.045	0.277	0.232, 0.287	0.259	4916	0.030	0.003, 0.058	0.030	0.024, 0.047	0.034	5219
10. Sleep duration	Beta	−0.009	−0.033, 0.014	0.426	0.392, 0.454	0.423	5127	−0.019	−0.042, 0.004	0.106	0.107, 0.149	0.127	5443
11. Behavioural difficulties	Beta	0.010	−0.021, 0.041	0.522	0.482, 0.544	0.513	5133	0.022	−0.008, 0.051	0.152	0.130, 0.176	0.152	5452
12. Depression	Beta	−0.006	−0.027, 0.015	0.557	0.524, 0.586	0.555	4885	−0.007	−0.027, 0.012	0.466	0.442, 0.504	0.473	5434
13. Sleep maintenance	OR	0.983	0.919, 1.051	0.589	0.534, 0.596	0.565	5127	0.973	0.913, 1.038	0.383	0.313, 0.372	0.342	5448
14. ODD	Beta	−0.004	−0.024, 0.016	0.700	0.683, 0.740	0.712	4943	0.015	−0.005, 0.034	0.148	0.146, 0.194	0.169	5319
15. Autism	OR	1.027	0.722, 1.460	0.874	0.860, 0.901	0.882	5975	1.153	0.803, 1.654	0.411	0.380, 0.442	0.411	6156
16. Sleep initiation	OR	0.995	0.934, 1.061	0.874	0.827, 0.873	0.851	5150	0.971	0.913, 1.032	0.309	0.269, 0.326	0.297	5476
17. Life events	Beta	<0.001	−0.018, 0.019	0.996	0.991, 0.999	0.997	5167	0.010	−0.008, 0.028	0.271	0.237, 0.292	0.264	5493

*Note*. The intergenerational analysis represents offspring phenotypes <10 years regressed on maternal GRS. The childhood analysis represents offspring phenotypes <10 years regressed on offspring GRS.

GRS = genetic risk scores; ODD = oppositional defiant disorder; ADHD = attention deficit hyperactivity disorder; IQ = intelligence quotient; BMI = body mass index.

#### Childhood GRS analyses

**Figure 3:**
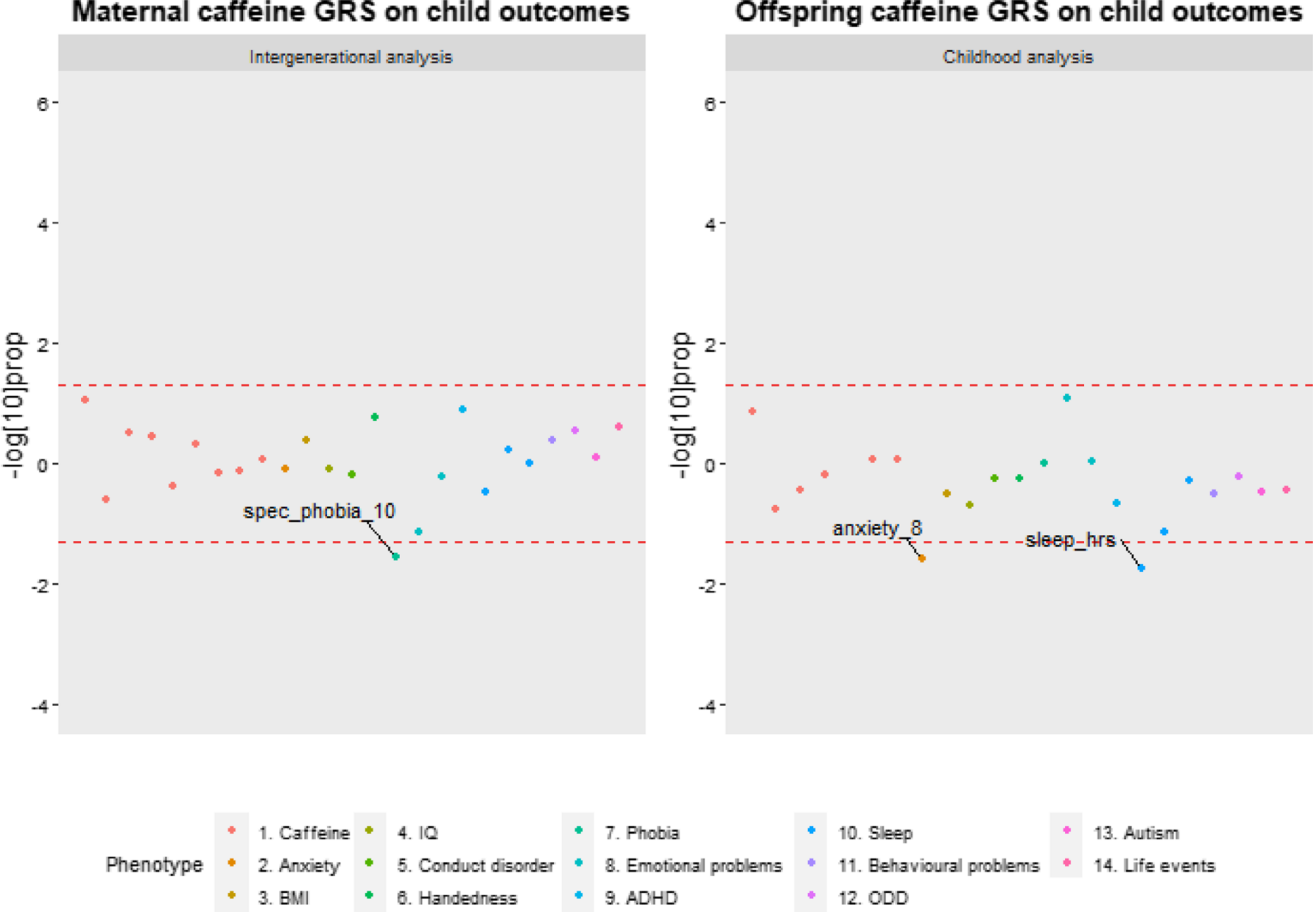
Comparison of phenotype associations with the caffeine genetic risk scores (GRS) in the intergenerational and childhood analysis. Points outside the lines had a permutation corrected *P* value < 0.05. Points above the upper line represent positive associations and points below the lower line represent negative associations. spec_phobia_10 = Specific phobias at age 10. anxiety_8 = Anxiety at age 8. sleep_hrs = Sleep duration in hours at age 7

As observed in the intergenerational analysis, there was some evidence for an association with reduced anxiety problems (P_perm_ = 0.002) and increased conduct disorder symptoms (P_perm_ = 0.001). In contrast to the intergenerational analysis, there was some evidence for an association with attention deficit hyperactivity disorder (ADHD symptoms) (P_perm_ = 0.034). The strongest non‐mental health associations of the intergenerational analysis were replicated using the offspring smoking GRS (lower IQ, P_perm_ < 0.001; increased caffeine consumption: P_perm_ = 0.048) with the exception of left‐handedness (P_perm_ = 0.291; Fig. 3). Only the associations with IQ and conduct disorder symptoms survived Bonferroni correction of *P* < 0.003 (Fig. 2, Table 4).

The results using lifetime smoking GRS were largely consistent. Only the associations with offspring's IQ, Oppositional Defiant Disorder (ODD) symptoms and total behavioural difficulties survived Bonferroni correction (**Supporting information Table** **S4** **)**.

### Comparison of intergenerational and childhood caffeine GRS analyses

#### Intergenerational GRS analyses

Given that offspring's caffeine GRS was not robustly associated with caffeine consumption in childhood (Table 3), we were able to use the results of the childhood analysis as a way of assessing pleiotropy despite some children already consuming low levels of caffeine at this age. Of the 17 childhood phenotypes, the strongest mental health association was observed with decreased risk for specific phobias in offspring (P_perm_ = 0.028; Fig. 3). There was no evidence for associations with any of the non‐mental health phenotypes.

#### Childhood GRS analyses

In contrast to the intergenerational analysis, there was no evidence for an association with specific phobias (P_perm_ = 0.998) but some evidence for an association with reduced general anxiety symptoms (P_perm_ = 0.026). The strongest association among the non‐mental health phenotypes was observed with fewer hours of sleep in term‐time (P_perm_ = 0.018), (Fig. 3 and Table 5). None of the associations of the intergenerational and childhood analyses for caffeine survived Bonferroni correction.

**Table 5:** Associations between maternal and offspring caffeine GRS and offspring phenotypes <10 years.

*Phenotype*	*Effect estimate*	*Intergenerational analyses*						*Childhood analyses*					
		*Regression analyses*			*Permutation testing*			*Regression analyses*			*Permutation testing*		
		*Effect size*	*95% CI*	*P value*	*95% CI*	*P value*	*Sample size*	*Effect size*	*95% CI*	*P value*	*95% CI*	*P value*	*Sample size*
1. Specific phobia	OR	0.724	0.519, 1.012	0.057	0.019, 0.040	0.028	5100	0.999	0.723, 1.381	0.997	0.993, 1.000	0.998	4900
2. Depression	Beta	−0.017	−0.039, 0.002	0.075	0.056, 0.089	0.071	4885	−0.017	−0.037, 0.002	0.081	0.055, 0.088	0.070	5434
3. ADHD	Beta	−0.018	−0.008, 0.050	0.161	0.110, 0.152	0.130	4916	−0.018	−0.046, 0.010	0.206	0.199, 0.251	0.224	5219
4. Handedness	OR	1.064	0.968, 1.169	0.178	0.143, 0.189	0.165	4849	0.980	0.897, 1.070	0.624	0.548, 0.610	0.579	5399
5. Life events	Beta	−0.008	−0.007, 0.030	0.228	0.217, 0.271	0.243	5167	−0.008	−0.027, 0.010	0.366	0.329, 0.390	0.359	5493
6. ODD	Beta	0.002	−0.032, 0.008	0.240	0.257, 0.314	0.285	4943	0.002	−0.018, 0.022	0.829	0.583, 0.644	0.614	5319
7. Sleep initiation	OR	0.972	0.913, 1.036	0.352	0.316, 0.375	0.345	5150	0.951	0.895, 1.010	0.094	0.059, 0.092	0.074	5476
8. Total caffeine	Beta	0.008	−0.015, 0.032	0.490	0.444, 0.506	0.475	4067	0.010	−0.012, 0.032	0.377	0.349, 0.410	0.379	4589
9. BMI	Beta	0.025	−0.033, 0.084	0.387	0.364, 0.425	0.394	5032	0.025	−0.027, 0.077	0.348	0.297, 0.356	0.326	5799
10. Behavioural difficulties	Beta	−0.015	−0.019, 0.043	0.441	0.369, 0.431	0.400	5133	−0.015	−0.044, 0.015	0.324	0.294, 0.353	0.323	5452
11. Emotional problems	Beta	0.001	−0.014, 0.027	0.538	0.569, 0.631	0.600	5139	0.001	−0.018, 0.021	0.883	0.881, 0.919	0.901	5459
12. Sleep duration	Beta	−0.026	−0.030, 0.017	0.577	0.544, 0.606	0.575	5127	−0.026	−0.048, −0.004	0.018	0.011, 0.028	0.018	5443
13. Conduct Disorder	Beta	−0.006	−0.024, 0.015	0.624	0.627, 0.686	0.657	5012	−0.006	−0.024, 0.013	0.563	0.541, 0.603	0.572	5326
14. Autism	OR	1.052	0.758, 1.461	0.742	0.733, 0.787	0.761	5975	0.850	0.603, 1.199	0.326	0.307, 0.366	0.336	6156
15. IQ	Beta	0.276	−0.521, 0.390	0.778	0.766, 0.817	0.792	4675	0.276	−0.155, 0.707	0.209	0.183, 0.234	0.208	5290
16. Anxiety	Beta	−0.022	−0.023, 0.019	0.849	0.805, 0.853	0.830	4993	−0.022	−0.042, −0.002	0.029	0.017, 0.038	0.026	5355
17. Sleep maintenance	OR	1.001	0.936, 1.071	0.970	0.947, 0.972	0.961	5127	0.983	0.922, 1.048	0.573	0.517, 0.579	0.548	5488

*Note.* The intergenerational analysis represents offspring phenotypes <10 years regressed on maternal GRS. The childhood analysis represents offspring phenotypes <10 years regressed on offspring GRS. ODD = oppositional defiant disorder; ADHD = attention deficit hyperactivity disorder; IQ = intelligence quotient; BMI = body mass index.

### Smoking and caffeine GRS analyses with phenotypes during and outside of pregnancy and during adolescence

The GRS for smoking were associated with these behaviours outside of pregnancy and during adolescence (Table 2). The caffeine GRS was associated with caffeine consumption outside of pregnancy (except for cola consumption) but not during adolescence (Table 3). The strongest evidence for associations with the smoking GRS was found for binge drinking, increased caffeine consumption, BMI and extraverted personality traits in mothers (Bonferroni threshold = 0.002) and more externalising problems and extraversion, increased BMI, and lower IQ in adolescence (Bonferroni threshold = 0.001). A detailed description of these results can be found in the Supporting Information and Supporting information Tables S5–S7. None of the caffeine GRS associations survived Bonferroni correction (**Supporting information Table** **S6** **)**.

## Discussion

In this study, we aimed to disentangle possible causal associations of maternal smoking and caffeine consumption, with a particular focus on the prenatal period, on offspring mental health from pleiotropic associations. Our results showed that the maternal smoking and caffeine GRS are valid predictors of smoking and caffeine consumption from tea and coffee during pregnancy. The maternal and offspring smoking initiation GRS were associated with various mental health traits and other substance use behaviours across different timepoints in life. In particular, we observed associations of the maternal and offspring smoking initiation GRS with sensation‐seeking traits across development, such as less anxiety and increased externalising problems in childhood, an extroverted personality type, more externalising problems and alcohol consumption in adolescence, as well as higher expression of anger, more monotony avoidance outside of pregnancy and alcohol consumption during and outside of pregnancy. We found few associations between the maternal and offspring caffeine GRS and offspring mental health outcomes. Critically, our results indicate that the associations found between the maternal smoking and caffeine GRS and offspring mental health outcomes are likely because of pleiotropic effects, rather than acting through the maternal intrauterine environment.

The literature supports our findings of pleiotropic associations between the maternal and offspring smoking GRS and sensation‐seeking personality traits. Previous studies found that adolescence who smoke have more externalising problems, higher impulsivity and novelty‐seeking type of behaviours [36] and that children with lower cognitive abilities have more behavioural problems and are more likely to initiate smoking themselves [37, 38]. There is evidence for shared genetic factors influencing smoking behaviours, externalising problems and novelty seeking type of behaviours [39, 40], as well as educational attainment [41]. However, some studies argue that the effect from the maternal postnatal environment (such as parenting behaviours) and maternal mental health cannot be dismissed even after accounting for genetic effects [42, 43]. We found some evidence that the maternal smoking GRS is associated with maternal depression during and outside of pregnancy, which could (partly) explain the association we observed between the maternal smoking GRS and offspring externalising problems. A study adopting a similar design to the present one, examining associations between maternal and offspring GRS for increased alcohol consumption and maternal and offspring mental health [44], also found an association between maternal alcohol use and maternal depression during pregnancy but no evidence for an association with maternal alcohol GRS and externalising problems in offspring. Even though this requires further testing, it could provide some initial evidence that the association between the maternal smoking GRS and offspring externalising problems is more likely to be pleiotropic than confounded by maternal depression. Further, other studies suggest that the genetic instrument for smoking initiation may not only measure smoking behaviour but also capture novelty‐seeking and impulsive behaviours even when only using genome‐wide significant SNPs [41, 45, 46, 47]. In addition, GSCAN summary statistics for smoking initiation showed a strong genetic correlation with ADHD and risk tolerance behaviour, which could make pleiotropic effects more likely [25]. Taken together with the existing literature, our findings support the notion that these observed associations with maternal smoking initiation GRS are likely explained by shared genetic liability in mothers and offspring.

We did not find strong evidence for intergenerational effects between the maternal caffeine GRS and offspring mental health outcomes in childhood. The associations we observed between maternal caffeine GRS and decreased likelihood of binge drinking, reduced caffeine consumption and lower socioeconomic position during pregnancy, as well as the offspring caffeine GRS and higher General Certificate of Secondary Education (GCSE) exam grades during adolescence stand in contrast to a study in the UK Biobank where the caffeine GRS was positively associated with alcohol consumption outside of pregnancy and not associated with social class [32]. Therefore, these findings should be interpreted with caution, because they might be unique to the ALSPAC sample in terms of participants’ sociodemographic characteristics or false positives. Although these results could be because of yet unexplained forms of bias, it is also possible that the caffeine GRS is capturing underlying personality/socio‐behavioural profiles with far reaching consequences for health and wellbeing, which deserves further investigation.

### Strengths and limitations

A major strength of this study was the exploration of exposure‐outcome associations at timepoints in life other than adulthood. Further, the validation of genetic variants discovered in non‐pregnant female and male populations, as proxies during pregnancy, is vital for future investigation of intrauterine effects of the exposures [24]. Last, the intergenerational comparison of associations of the maternal smoking and caffeine GRS with childhood mental health outcomes that are likely to be free of confounding through offspring's own substance consumption enabled us to disentangle potential pleiotropic and environmental effects on mental health.

The following limitations should also be considered. First, the limited sample size (in the context of genetic association studies) likely resulted in low statistical power to detect small effects. Second, we were restricted to phenotypes as assessed in ALSPAC, and the comparison of related phenotypes was not similar across development (e.g. ADHD/conduct disorder in childhood with extraversion and anger personality traits in mothers outside of pregnancy). Third, many mental health phenotypes in childhood were based on maternal report, which may not accurately reflect offspring's mental health problems [48, 49] but rather mothers own mental health status [50, 51]. Fourth, we constructed GRS for smoking initiation based on the latest GWAS that included ALSPAC mothers [25]. Because of the sample overlap, the true strength of explored associations might be smaller than we reported. However, given the small contribution of data from ALSPAC (~1%) to a total sample size of 1.2 million, the risk of bias is likely negligible. Fifth, to make the smoking GRS specific to our exposure of interest we based our GRS on genome‐wide significant SNPs only, yet the smoking GRS still showed associations with some alcohol phenotypes. We checked the correlations between the alcohol, smoking and caffeine GRS, which were low (**Supporting information Table** **S8** **)**. However, because of the phenotypic associations with alcohol consumption, we cannot rule out that associations observed with the maternal smoking GRS are cofounded by maternal alcohol consumption. Still, this is unlikely to affect our results because we did not find evidence for potential causal effects, and previous research by Easey and colleagues observed no associations in intergenerational analyses between maternal alcohol GRS and offspring mental health outcomes [44]. Sixth, because our dataset included phenotypes from later timepoints and we relied on participants whose genotype data was available, it is possible that our findings are subject to selection bias [31, 52]. Last, the comparison of the intergenerational and childhood GRS analyses was based on transmitted alleles and therefore an indirect effect of maternal non‐transmitted alleles on offspring sensation‐seeking traits through genetic nurturing cannot be ruled out [53].

### Future research

Future studies investigating the effects on mental health using the smoking initiation GRS might consider accounting for sensation seeking personality traits. Further, future research should aim to differentiate effects of smoke exposure through the intrauterine and postnatal environment, explore non‐linear effects of the smoking and caffeine GRS and investigate a potential interaction of smoking and caffeine consumption during pregnancy on offspring mental health [54]. More analyses exploiting paternal data would be helpful to understand the effect of smoking and caffeine consumption on offspring mental health outcomes. For instance, studies with paternal genotype data could help to differentiate whether observed effects are because of intrauterine or postnatal exposure, through conducting negative control comparisons of prenatal associations of maternal and paternal substance use.

## Conclusion

In conclusion, our study validated the application of the smoking initiation, lifetime smoking and caffeine GRS for research investigating intrauterine exposures to smoking and caffeinated coffee and tea. Further, we found stronger evidence for pleiotropic rather than causal effects of maternal smoking and caffeine consumption on offspring mental health. Given the current study's limitations, particularly its limited statistical power, these findings should be replicated in independent samples using more refined methods for pleiotropy detection and corrections.

## Declarations of interests

None.

## Funding

This research was performed in the UK Medical Research Council Integrative Epidemiology Unit (grant number: MC_UU_00011/7) and also supported by the National Institute for Health Research (NIHR) Bristol Biomedical Research Centre at University Hospitals Bristol National Health Service (NHS) Foundation Trust and the University of Bristol. The Medical Research Council (MRC) also funded K.E.E.'s PhD studentship. L.Z. was supported by a UK MRC fellowship (grant number G0902144). G.C.S. was supported by the MRC (New Investigator Research Grant, MR/S009310/1) and the European Joint Programming Initiative ‘A Healthy Diet for a Healthy Life’ (JPI HDHL, NutriPROGRAM project, UK MRC MR/S036520/1).

This research was also conducted as part of the Childhood and Adolescence Psychopathology: unravelling the complex aetiology by a large Interdisciplinary Collaboration in Europe (CAPICE) project, funded by the European Union's Horizon 2020 research and innovation programme, Marie Sklodowska Curie Actions–MSCA‐ITN‐2016–Innovative Training Networks under grant agreement number 721567. This study was supported by the NIHR Biomedical Research Centre at the University Hospitals Bristol NHS Foundation Trust and the University of Bristol. The views expressed in this publication are those of the authors and not necessarily those of the NHS, the NIHR or the Department of Health and Social Care.

The views expressed in this publication are those of the authors and not necessarily those of the NHS, the NIHR or the Department of Health.

A comprehensive list of grant funding is available on the ALSPAC website (http://www.bristol.ac.uk/alspac/external/documents/grant‐acknowledgements.pdf). GWAS data was generated by Sample Logistics and Genotyping Facilities at Wellcome Sanger Institute and LabCorp (Laboratory Corporation of America) using support from 23andMe.

## Author contributions

**Laura Schellhas:** Conceptualization; data curation; formal analysis; methodology; project administration; visualization. **Elis Haan:** Conceptualization; data curation; formal analysis; methodology; project administration; visualization. **Kayleigh Easey:** Conceptualization; data curation; formal analysis; methodology; project administration. **Robyn Wootton:** Conceptualization; data curation; formal analysis; methodology; project administration; supervision; visualization. **Hannah Sallis:** Conceptualization; data curation; formal analysis; methodology; project administration; supervision. **Gemma Sharp:** Conceptualization; funding acquisition; methodology; supervision. **Marcus Munafo:** Conceptualization; formal analysis; funding acquisition; methodology; project administration; supervision. **Luisa Zuccolo:** Conceptualization; data curation; formal analysis; funding acquisition; methodology; project administration; supervision; visualization.

## Supporting information


**Table S1** List of phenotypes included in the study.
**Table S2** Comparison of participants with complete and partially missing genotype data.
**Table S3** Associations between the lifetime smoking GRS and smoking phenotypes in mothers during and outside of pregnancy and adolescents.
**Table S4** Associations between maternal and offspring lifetime smoking GRS and offspring phenotypes <10 years.
**Table S5** Associations between the maternal and offspring smoking initiation GRS and phenotypes in mothers during and outside of pregnancy and adolescence.
**Table S6** Associations between maternal and offspring lifetime smoking GRS and phenotypes in mothers during and outside pregnancy and adolescence.
**Table S7** Associations between caffeine GRS and phenotypes in mothers during and outside of pregnancy and adolescence.
**Table S8** Correlation between smoking, caffeine and alcohol GRS.
